# A pharmacovigilance system for treatment access and medical donation programs: The Max Foundation experience

**DOI:** 10.1186/s12992-018-0391-4

**Published:** 2018-07-27

**Authors:** Ann Kim Novakowski, Pat Garcia-Gonzalez, Michael Wrigglesworth, Andy Stergachis

**Affiliations:** 1 The Max Foundation, 200 NW Pacific St, Suite 103, Seattle, WA 98105 USA; 2 Global Medicines Program, Schools of Pharmacy and Public Health, University of Washington, BOX 357631, Seattle, WA 98195-7631 USA

**Keywords:** Pharmacovigilance, Medical donations, Low- and middle-income countries, Imatinib, Patient assistance programs, Drug safety

## Abstract

**Background:**

Cancer is a major burden of disease in low- and middle-income countries (LMICs) yet financial barriers limit access to life-saving oncology drugs. Medical donation and other drug access programs can help improve patient access to essential medicines, such as quality assured oncology drugs in LMICs. However, there are no published examples of the conduct of pharmacovigilance with donated medical products intended for use in LMICs where pharmacovigilance is weak. We describe a partnership between a pharmaceutical company and a non-governmental organization as a case example that addresses the challenges in performing pharmacovigilance with donated medicines in LMICs. The Max Foundation’s direct to patient model is designed to improve global access to quality assured oncology drugs through access programs such as the Glivec® (generic name: imatinib) International Patient Assistance Program (GIPAP).

**Results:**

Between 2013 and 2016, in the course of managing the GIPAP program, The Max Foundation was made aware of 13,039 instances of adverse events (AEs). These AEs were reported to The Max Foundation by physicians, patients, and caregivers. The Max Foundation reported these AEs to Novartis through the AE reporting tool within its Patient Assistance Tracking System (PATS). Physicians were the reporters for 58% of the AEs while the remainder of the AEs were reported directly by patients or caregivers. The overall rate of reported AEs remained relatively steady for the years 2013 through 2016 at 92, 95, 86, and 97 AEs reported per 1000 persons who received Glivec® per year, respectively. The vast majority of adverse events (85%) were reported from countries where The Max Foundation has a MaxStation, i.e., where The Max Foundation staff interact directly with physicians and patients at clinics or over the phone. AE reporting rates were consistently higher in all years studied from countries where The Max Foundation has a MaxStation. While India accounted for the largest number of reported adverse events in 2016 (1990), Bolivia had the highest rate of reported adverse events at 484 AEs per 1000 patients.

**Conclusions:**

International patient assistance programs that provide access to medicines can have an important role in assisting pharmaceutical companies in fulfilling their pharmacovigilance obligations. Adverse event information collected through PATS can potentially contribute to the overall body of knowledge on the safety of medicinal products.

## Background

Medical product donation programs and other treatment access programs can address multiple needs that arise in a variety of situations, including health system strengthening in low- and middle-income countries (LMICs). Pharmaceutical companies alone donate drugs valued at an estimated 3.8 billion USD annually [1]. While medical donation programs for neglected tropical diseases (NTDs) and other communicable diseases are more common, donors are increasingly providing access to medicines to address the burden of non-communicable diseases (NCDs) in LMICs, such as cancer. Cancer is one of the leading causes of morbidity and mortality worldwide. In 2016, there were 17.2 million incident cancer cases, 8.9 million deaths, and 213.2 million DALYs due to cancer globally [2]. There are a number of challenges to reducing the burden of cancer in LMICs, including the lack of diagnostic and treatment capacity and scarce and expensive oncology medicines.

It is essential for medical product donation and other access programs to include provisions to ensure patient safety. To help ensure patient safety with medical products, medicine regulatory agencies, such as the US Food and Drug Administration (FDA) and the European Medicines Agency (EMA), routinely receive and analyze information on adverse events (AEs) to drugs, vaccines, and medical devices during the post-approval period. An AE is “any untoward medical occurrence in a patient or clinical investigation subject administered a pharmaceutical product and which does not necessarily have a causal relationship with this treatment,” whereas an adverse drug reaction is “a response to a drug which is noxious and unintended and which occurs at doses normally used in man for prophylaxis, diagnosis, or therapy of disease or for modification of physiological function.” [3]

As pharmaceutical companies and others make oncology medicines available to patients in LMICs, they are responsible for including provisions that monitor and evaluate patient safety on a continuous basis. Market authorization holders (MAH), i.e., pharmaceutical companies, are legally required to collect, investigate, and submit reports of adverse events concerning their products, regardless of country of occurrence. For example, the EMA stipulates that marketing authorization holders in the European Union (EU) are required to report all suspected adverse reactions occurring in the EU and in other markets [4]. And in the US, pharmaceutical companies, health care professionals, and consumers submit reports of suspected adverse reactions to the FDA for domestic as well as foreign adverse events associated with FDA-regulated drug products. In 2014, of the 1,204,685 reports contained in that year’s FDA Adverse Event Reporting System (FAERS) database, 31% were foreign reports [5]. While safety surveillance is not specifically called out in the WHO Guidelines for Medical Donations, evaluations are recommended to assess appropriateness of medical donations [6]. The Partnership for Quality Medical Donations (PQMD), recognizing the importance of safety reports, published guidelines for medical donation programs that include the need for member organizations to have adverse event reporting procedures [7].

Despite such requirements and recommendations, we could find no published examples whereby pharmacovigilance was systematically performed by pharmaceutical companies or by non-governmental organizations with donated medical products used in LMICs where pharmacovigilance systems are weak. Assessments of pharmacovigilance systems in LMICs have identified gaps indicating many LMICs have minimally functional pharmacovigilance systems, including low rates of reporting of AEs [8–10]. Moreover, LMICs’ national pharmacovigilance programs can lack an adequate understanding of the role that multinational pharmaceutical companies can play in the conduct of routine pharmacovigilance for their products as well as the considerable level of pharmacovigilance expertise within many multi-national pharmaceutical companies and certain non-governmental organizations (NGOs). There is a need for improving the evidence-base on innovative approaches to global pharmacovigilance. Working together, NGOs and MAH have the potential for improving AE reporting, thereby fulfilling MAHs legal obligations and potentially contributing to strengthening national pharmacovigilance systems. The objective of this manuscript is to describe a novel pharmacovigilance program developed by an NGO and illustrate its performance in terms of number of reported AEs to a MAH for a donated product in LMICs.

## Methods

The largest international oncology patient access program, the Glivec® International Patient Assistance Program (GIPAP), was developed by Novartis to ensure patients with cancer receive treatment, e.g., chronic myeloid leukemia (CML) and metastatic malignant gastrointestinal stromal tumors (GIST) [11]. GIPAP was established in 2001 by Novartis Pharma AG and implemented in partnership with The Max Foundation, a USA-based, non-profit, international NGO [12]. Since that time, GIPAP expanded to a global network of more than 75 LMICs, reaching 75,000+ patients at no cost to eligible patients in those countries [13]. Briefly, patient eligibility is based on diagnosis with Philadelphia chromosome positive chronic myeloid leukemia (Ph + CML) and patients with c-Kit (CD117) positive, inoperable and/or metastatic malignant gastrointestinal stromal tumors (GIST) as well as on financial need, i.e., patients who are not insured or reimbursed, cannot pay for treatment privately, and live in countries that have minimal reimbursement capabilities for their condition. GIPAP provides Glivec® at no cost to patients. Out-of-pocket costs, such as diagnosis and travel to health visits, are not covered by Novartis or The Max Foundation. The Max Foundation’s role in GIPAP is to receive applications from qualified physicians on behalf of individual patients, verify that each patient fulfills program criteria, and approve each application. In addition, The Max Foundation coordinates between physicians and the company for the availability of treatment for each patient. Given that Glivec® is a long-term treatment, The Max Foundation requests that physicians confirm treatment continuation every four (4) months for each individual patient. The organization also provides information and referral assistance to patients, their families, and caregivers.

### The Max Foundation’s Patient Assistance Tracking System (PATS)

The Max Foundation developed the Patient Assistance Tracking System (PATS), a proprietary, in-house, web-based, customer relationship management tool, to support and coordinate all of the organization’s activities in GIPAP, including the recording and reporting of all AEs that they become aware of from physicians, patients, or caregivers within GIPAP [10]. All team members with an active role in GIPAP have access to PATS. Qualified and approved physicians also have access to PATS, through a secure login, to provide The Max Foundation team with updated treatment information for their patients in GIPAP.

As a proxy of Novartis, The Max Foundation reports all adverse events to Novartis that they become aware of occurring within GIPAP. Following each AE report submitted by The Max Foundation, the Novartis Safety team works with each treating physician to complete any additional information that may be required and submits AE reports to health authorities as per their specific regulatory reporting requirements. The GIPAP agreement between The Max Foundation and approved institutions/physicians specifies that the qualified institution/physician should report adverse events as required by their national legislation. Further, the agreement states that The Max Foundation: “will report to Novartis all AEs that they learn of through the participation of a patient in the GIPAP program regardless of the seriousness or suspectedness of the event. This reporting is separate from the local reporting obligation that physicians must adhere to based on their local reporting requirements. Novartis will direct all follow-up questions to the GIPAP physician (including for reports received from patients and/or caregivers) and will submit AE reports to Health Authorities as required.”

The Max Foundation’s pharmacovigilance activities are supported by the organization’s standard operating procedures along with PATS. The Max Foundation staff undergoes training on AE reporting and performs quality control of reported AEs in accordance with established policies and procedures. Initial and annual training is performed by a Novartis drug safety representative for all staff at The Max Foundation involved in GIPAP. The AE reporting training modules include information on the importance of reporting, what should be reported to Novartis, AE definitions, timelines for reporting, and monitoring activities to ensure AEs are reported completely and accurately. In addition to annual training performed by Novartis, The Max Foundation undergoes mid-year training for all team members involved in GIPAP to ensure that there is clarity on the process and requirements for reporting. This includes training team members to provide narrative and contextual information and word-for-word reports, as relevant, by the reporter. In some cases, reports are also submitted in the same language as the reporter.

The PATS system is designed to extract all pertinent fields and narrative information to construct each AE report and send these reports to the Novartis Patient Safety organization. In the case of AEs reported by physicians into the PATS system, the system automatically submits all physician-reported AEs immediately and in real time to the Novartis Safety Desk. AE reports received verbally or in writing, from any reporter are entered manually by The Max Foundation’s team into PATS within 24 hours of receiving the report. The PATS system forwards them to the Novartis Safety Desk immediately. All reports submitted in PATS are retained indefinitely. A reporting feature within PATS enables the team to reconcile reports submitted by The Max Foundation and received by Novartis as well as perform quality control of reports against the original source documents on an annual basis.

**Fig. 1:**
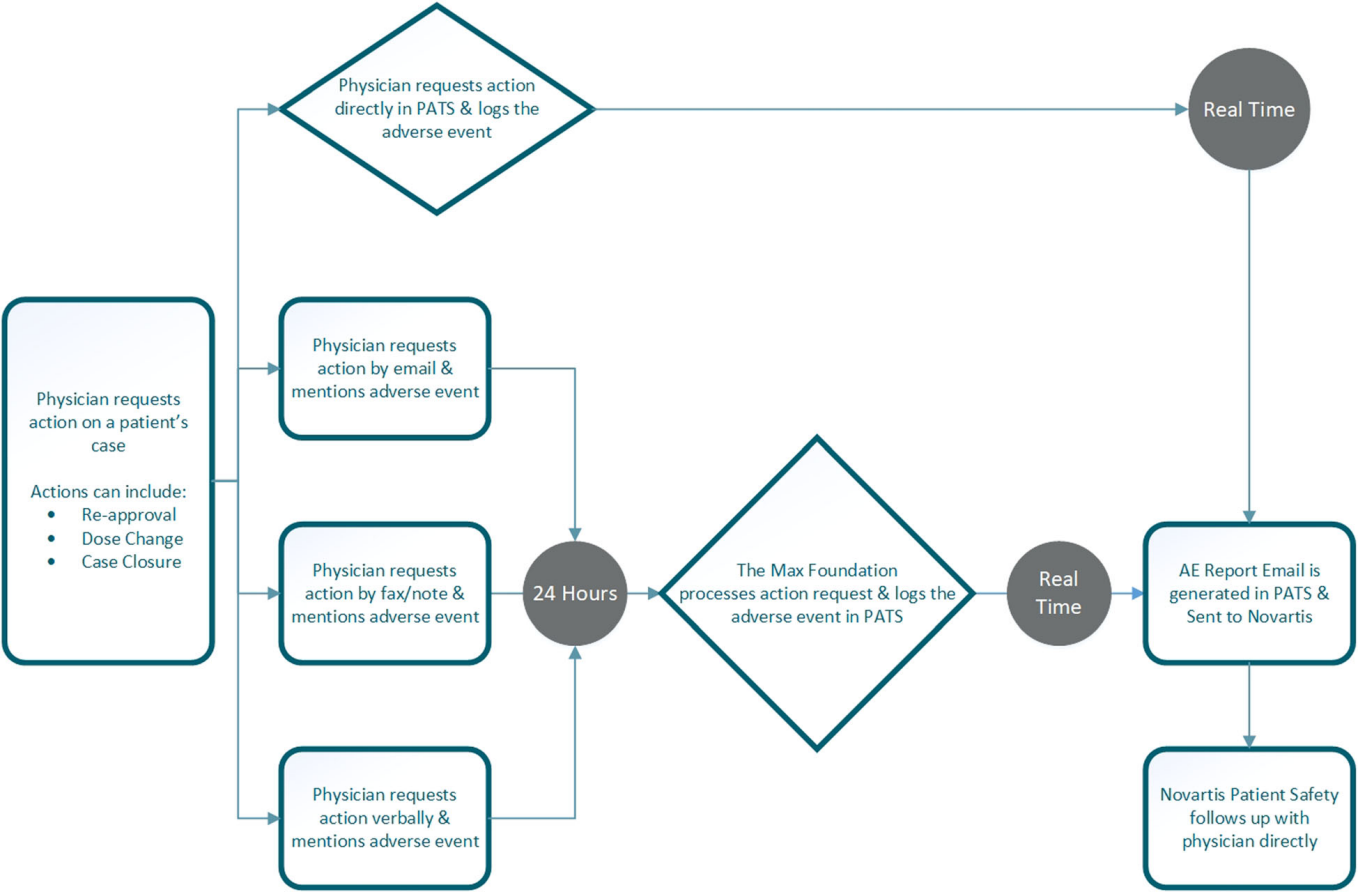
Adverse Event Report Flowchart for the Glivec® International Patient Assistance Program (GIPAP) Where the Reporter is a Physician

**Fig. 2:**
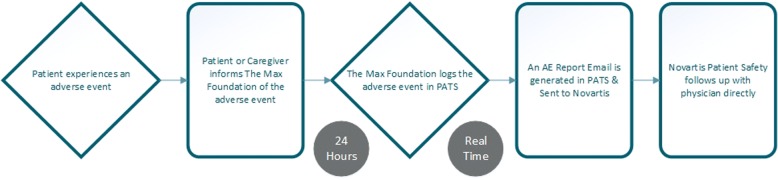
Adverse Event Report Flowchart for the Glivec® International Patient Assistance Program (GIPAP) Where the Reporter is the Patient or a Caregiver

**Fig. 3:**
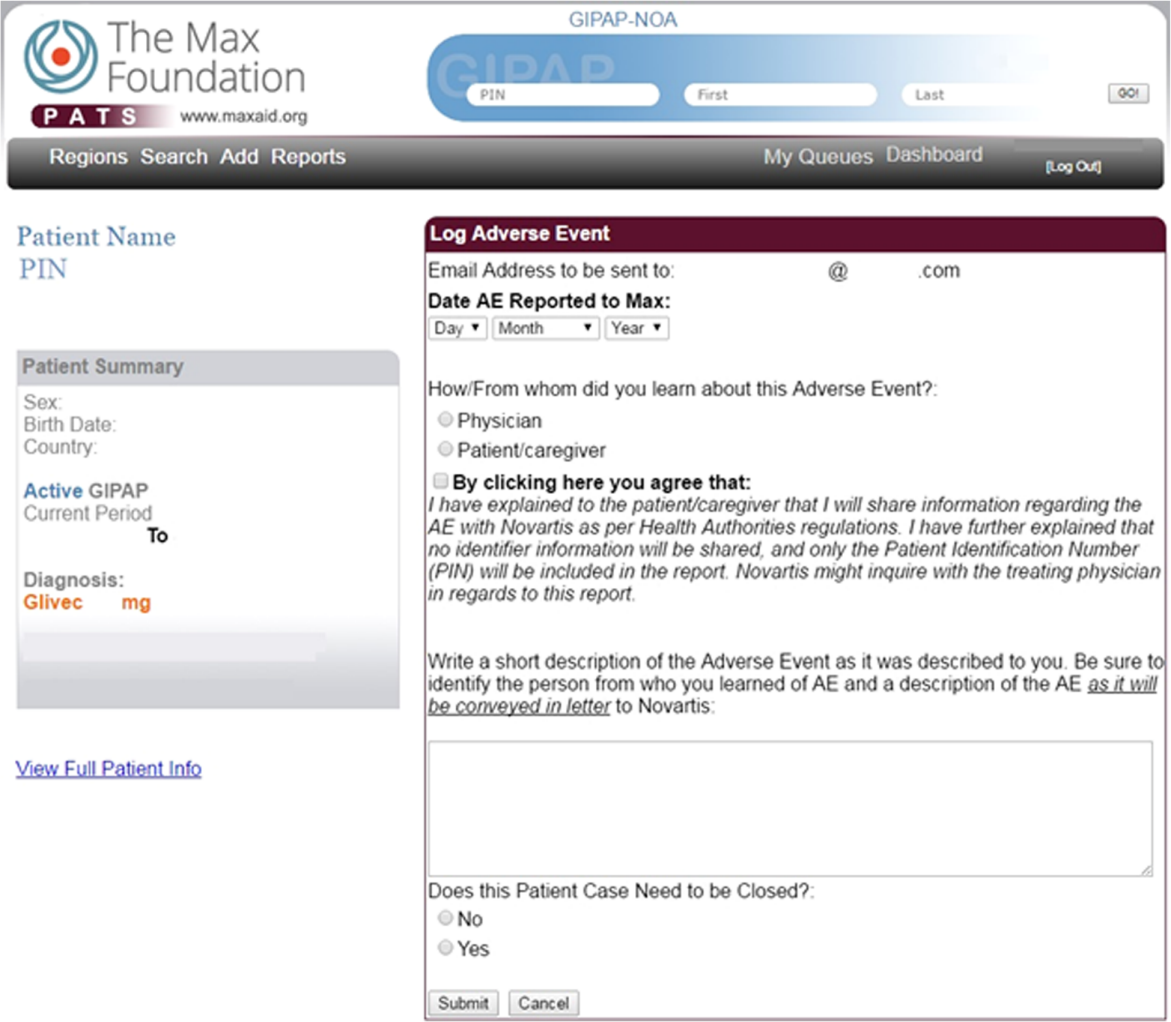
Screenshot of Patient Assistance Tracking System as Viewed by The Max Foundation Staff

Figure 1 depicts the flow of AE reporting when the reporter is physician while Fig. 2 describes the flow when the reporter is a patient or a caregiver. Figure 3 is an illustrative screenshot of PATS.

### Analysis

Counts of adverse events reported through PATS were obtained for the 4-year period of January 1, 2013 through December 31, 2016. Additionally, the number of patients receiving Glivec® annually through GIPAP was obtained for this time period. Data were analyzed by year, country, reporter (physician or patient/caregiver), and whether or not the country had a MaxStation, i.e., where The Max Foundation staff interact with physicians and patients at clinics, including where MaxStation staff call patients for follow-up. Descriptive statistics are expressed as counts, percentages, or rates per 1000 patients who received Glivec® through GIPAP. The Chi square test was performed to determine if there was an association between AE reporting by physicians and whether or not the country had MaxStation.

## Results

**Fig. 4:**
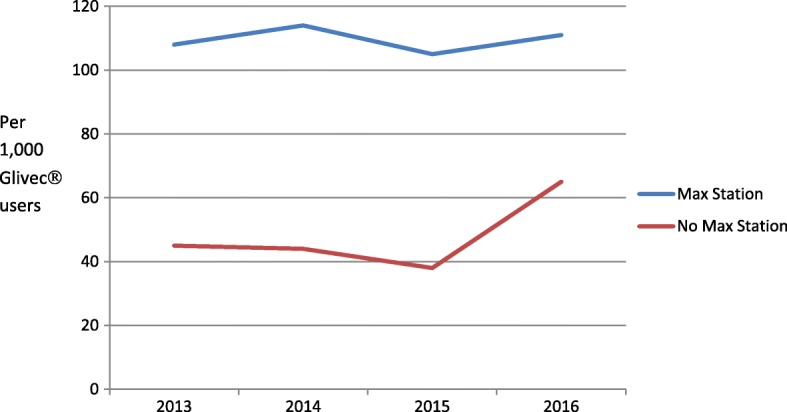
Rate of adverse event reports per 1000 Glivec® users by year and MaxStation

Between 2013 and 2016, a total of 13,039 AEs were reported through The Max Foundation’s Patient Assistance Tracking System (Tables 1 and 2). The vast majority of AEs (85%) were reported from countries where The Max Foundation has a MaxStation. Additionally, rates of AE reports per 1000 Glivec® users were consistently higher in all years studied from countries where The Max Foundation has a MaxStation (Fig. 4). Among countries with a MaxStation, physicians were the reporters for 60% of AEs (*n* = 6679 AEs) whereas physicians were reporters for 83% of the AEs (*n* = 1605) among countries without a MaxStation. In contrast, 40% of the AEs (*n* = 4421) were reported directly by patients or caregivers among countries with a MaxStation while only 17% (*n* = 334) of AEs were reported directly by patients or caregivers among countries without a MaxStation, Chi square = 364, *p* < 0.0001. The overall rate of reported AEs remained relatively steady for the years 2013 through 2016 at 92, 95, 86, and 97 AEs reported per 1000 persons who received Glivec® through GIPAP per year, respectively. While India accounted for the largest number of reported adverse events in 2016 (1990), Bolivia had the highest rate of reported adverse events at 484 AEs per 1000 patients (Table 3).

**Table 1:** Number of Glivec® users and adverse event reports by year from countries with a Max-Station*

Country	2013		2014		2015		2016	
	No. of Users	AE Reports	No. of Users	AE Reports	No. of Users	AE Reports	No. of Users	AE Reports
India	18,003	1669	18,434	1821	19,117	1839	18,210	1990
Thailand	2096	331	2255	317	2496	207	2620	238
South Africa	756	128	851	163	727	95	604	40
Mexico	885	97	937	106	889	110	822	71
Chile	185	89	198	77	220	61	245	78
Uzbekistan	701	68	767	65	787	119	973	41
Bolivia	159	54	173	34	197	60	223	108
Philippines	322	40	297	67	271	41	258	33
Guatemala	201	36	224	39	255	40	273	39
Honduras	184	27	213	60	224	35	253	47
Paraguay	110	27	102	21	108	18	118	25
Jamaica	83	17	87	14	86	18	96	18
Ecuador	38	12	32	7	28	11	25	7
Malaysia	218	12	247	44	266	30	261	20
Argentina	58	11	56	9	54	9	50	12
El Salvador	151	10	161	14	174	10	177	24
Dominican Republic	137	7	115	13	111	10	104	7
Nicaragua	69	5	82	10	104	13	120	20
Peru	59	5	58	10	52	10	45	7
Bahamas	6	1	5	0	6	0	7	1
Total	24,422	2646	25,294	2891	26,172	2736	25,484	2826

^*^Countries with a Max-Station (MS) include those countries where MS call the patients for follow up and otherwise interact with the physician

**Table 2:** Number of Glivec® users and adverse event reports by year from countries without a Max-Station

Country	2013		2014		2015		2016	
	No. of Users	AE Reports	No. of Users	AE Reports	No. of Users	AE Reports	No. of Users	AE Reports
Azerbaijan	355	88	406	40	456	24	350	0
Vietnam	1294	47	1507	87	1631	68	1719	92
Sudan	1123	44	1266	66	1181	31	1338	113
Armenia	189	21	208	8	227	11	251	1
Kenya	553	20	658	25	788	36	914	114
Georgia	259	19	271	6	288	8	337	10
Nepal	852	11	980	13	1103	20	1260	29
Madagascar	17	10	40	3	52	7	59	1
Sri Lanka	469	10	454	6	421	12	390	6
Kazakhstan	125	9	123	22	112	4	61	9
Ghana	88	8	104	12	125	8	159	1
Mauritius	40	8	48	7	59	12	63	14
Republic of Congo	32	8	39	8	37	6	47	4
Nigeria	574	7	673	13	767	14	907	68
Cambodia	48	5	54	7	69	15	83	5
Malawi	7	5	7	2	20	6	28	1
Morocco	144	5	138	4	132	3	129	5
Ethiopia	471	4	573	3	721	6	858	63
Haiti	15	4	23	0	23	2	23	2
Kyrgyzstan	126	4	135	5	178	3	194	6
Moldova	95	4	101	8	107	8	130	13
Pakistan	590	4	546	2	0	0	0	0
Tanzania	94	4	105	2	105	1	146	16
Zimbabwe	89	4	117	3	148	6	176	12
Burkina Faso	22	3	25	1	39	1	54	2
Cameroon	79	3	99	4	101	5	113	3
Rwanda	53	3	51	3	65	3	85	12
Solomon Islands	5	3	4	4	2	0	5	0
Surinam	14	3	15	2	17	1	20	2
Zambia	38	3	47	6	58	2	64	1
Benin	28	2	37	5	32	5	45	10
Gabon	21	2	23	3	26	0	29	1
Indonesia	128	2	109	1	106	9	97	6
Niger	9	2	14	0	21	5	23	6
Uganda	169	2	185	2	218	2	252	23
Belarus	20	1	19	0	19	0	19	0
Central Africa Republic	3	1	1	1	1	0	1	0
Cote d’Ivoire	78	1	113	17	121	0	129	2
Guinea	2	1	2	2	2	1	0	0
Mali	56	1	78	2	81	4	95	5
Mongolia	48	1	58	16	64	5	70	3
Saint Lucia	7	1	8	1	10	0	10	2
Senegal	128	1	148	5	190	22	219	49
Sierra Leone	2	1	2	0	2	1	2	0
Bangladesh	3	0	3	0	3	0	3	0
Bhutan	21	0	26	0	27	1	25	0
Democratic Republic of Congo	4	0	4	0	9	1	18	7
East Timor	3	0	3	0	3	0	6	0
Fiji	12	0	11	1	16	3	19	1
Namibia	13	0	16	0	20	0	19	4
Papua New Guinea	30	0	30	2	37	1	52	2
Seychelles	8	0	8	1	8	3	7	3
Togo	50	0	57	1	59	2	50	0
Total	8703	390	9772	432	10,107	388	11,153	729

**Table 3:** Number and rate of adverse event reports by country for countries with a Max-Station, 2016

Country	Number of Glivec Users	Total AE Reports	Number of AE Reports per 1000 Glivec Users
Bolivia	223	108	484
Chile	245	78	318
Ecuador	25	7	280
Argentina	50	12	240
Paraguay	118	25	212
Jamaica	96	18	188
Honduras	253	47	186
Nicaragua	120	20	167
Peru	45	7	156
Guatemala	273	39	143
Bahamas	7	1	143
El Salvador	177	24	136
Philippines	258	33	128
India	18,210	1990	109
Thailand	2620	238	91
Mexico	822	71	86
Malaysia	261	20	77
Dominican Republic	104	7	67
South Africa	604	40	66
Uzbekistan	973	41	42
Total	25,484	2826	111

## Discussion

The Max Foundation experience indicates that the training, procedures, and systems established by the organization led to establishing a pharmacovigilance system that recorded and reported AEs from physicians and patients/caregivers to the market authorization holder. Initial and ongoing staff training emphasizes AE reporting importance, the type of information to report, and timelines for reporting. The Max Foundation procedures delineate roles and responsibilities on AE recording and reporting for this NGO, participating institutions/physicians and the MAH. Moreover, the AE reporting tool within PATS ensured timely reporting, as well as tracking and retention of AE reports.

The highest number of reported adverse events was from those countries with a MaxStation, most likely due to a combination of factors, including higher use of Glivec® through GIPAP in those countries as well as regular interactions among The Max Foundation staff, patients, and physicians. Others have cited the importance of ongoing interactions with focal persons in promoting reporting of adverse events [14, 15]. Effective communication helps to overcome the near-universal problem of underreporting of AEs. Notably, 4755 of the AE reports filed by The Max Foundation were from patients or caregivers. While uncommonly assessed in LMICs, these sources of experiences with the safety of oncology medications help understand patients’ perspectives, such as the effects of medications on quality-of-life [16].

India had the highest volume of AE reports. The volume of reports in India is due, in part, to the high volume of patients enrolled in the program. Over the reporting period, patients receiving Glivec® in India accounted for approximately 50% of all patients enrolled globally each year among all countries with a MaxStation. As a result of high enrollment, The Max Foundation’s team in India also account for more than half of all staff, enabling patients to have more touch points with a member of the team. Such touch points are critical for patients who have questions about doctor’s appointments, how to take the medication, what to do in the case of side effects, and attending patient meetings to connect with other patients and learn more about their disease.

Pharmacovigilance of oncology medicines poses certain challenges that many LMICs may be ill-equipped to properly address. The potentially high toxicity and narrow therapeutic window of many oncology drugs makes pharmacovigilance essential in oncology. Moreover, frequent use of multiple treatment regimens makes it difficult to disentangle the side effects of individual as well as combinations of medicines. Evaluation is complicated further due to many patients likely having significant disease-related morbidity. Finally, lifelong treatment with certain oncology medications, such as Glivec®, makes it likely that patients may experience AEs during their extended course of treatment. Based on findings presented here, training, procedures, and technology provided by The Max Foundation can improve drug safety reporting in LMICs.

This analysis has limitations. Our analysis does not characterize the types of AEs reported through PATS as this was beyond the scope of our study and beyond the scope and expertise of most NGOs. Rather, our aim was to demonstrate that AE reporting for medical donation programs is feasible through well-planned and coordinated efforts between the NGO and the MAH involving training, procedures, agreements, and systems. We also do not know the extent to which AEs from this program led to specific risk management or risk communication actions either on the part of the company or any of the national pharmacovigilance programs. There is anecdotal information of concerns raised by a few of the donation recipient countries’ health authorities, due to the high volume of AE reports in the access programs managed by The Max Foundation, while it is evident that other access programs under-report AEs. This creates an artificial bias towards potential safety concerns being highlighted for Glivec®. Nevertheless, reporting of AEs is recognized as a cornerstone of pharmacovigilance by efficiently generating safety signals and better characterizing at-risk groups, risk factors, and clinical characteristics of AEs. Spontaneous reported AEs can be evaluated and may be acted on for risk management and communication even for medicines, such as Glivec®, that have been marketed for use for many years and whose common adverse events are known.. Finally, we do not know whether or not AE reporting rates for Glivec® in these LMICs begin to achieve the level AE reporting rates for this drug in high-income countries. However, it is likely that the AE reporting rates achieved through GIPAP are higher than those that are found for this or most other medicines in LMICs. For example, AE reporting rates to NMRAs have been reported to be low by Aagaard et al., and by Ampadu et al. [17, 18] Both of these publications cite reporting rates for individual case safety reports (ICSRs) per million inhabitants per country.

In 2017 The Max Foundation and Novartis renewed their commitment to patients by launching CMLPath to Care™ a collaboration aimed at providing humanitarian access to treatment for CML and other rare cancers for those patients for whom no other form of national treatment access exists today [12]. CMLPath to Care™ consists of humanitarian product donations and funding support from Novartis to The Max Foundation, and an innovative new distribution model developed and implemented by The Max Foundation and its international distributor, under the Max Access Solution umbrella, replacing the Glivec® International Patient Assistance Program. The organization’s commitment to pharmacovigilance continues through The Max Foundation’s operational program, Max Access Solutions (MAS). Further, in 2017 The Max Foundation receives product donation from four other major drug manufacturers and utilizes PATS and its AE reporting tool to track and report all AEs they become aware of.

## Conclusion

Patient assistance programs that provide access to medicines can have an important role in global pharmacovigilance by collecting AE reports and submitting them to market authorization holders. Such real world data has the potential for informing the ongoing evaluation of the safety profile of donated marketed drugs. Future studies should examine the contribution of AE reporting performed by drug donation programs to strengthening the pharmacovigilance systems of LMICs.
